# From big data analysis to personalized medicine for all: challenges and opportunities

**DOI:** 10.1186/s12920-015-0108-y

**Published:** 2015-06-27

**Authors:** Akram Alyass, Michelle Turcotte, David Meyre

**Affiliations:** Department of Clinical Epidemiology and Biostatistics, McMaster University, 1280 Main Street West, Hamilton, ON Canada; Department of Pathology and Molecular Medicine, McMaster University, 1280 Main Street West, Hamilton, ON Canada

**Keywords:** Big data, Omics, Personalized medicine, High-throughput technologies, Cloud computing, Integrative methods, High-dimensionality

## Abstract

Recent advances in high-throughput technologies have led to the emergence of systems biology as a holistic science to achieve more precise modeling of complex diseases. Many predict the emergence of personalized medicine in the near future. We are, however, moving from two-tiered health systems to a two-tiered personalized medicine. Omics facilities are restricted to affluent regions, and personalized medicine is likely to widen the growing gap in health systems between high and low-income countries. This is mirrored by an increasing lag between our ability to generate and analyze big data. Several bottlenecks slow-down the transition from conventional to personalized medicine: generation of cost-effective high-throughput data; hybrid education and multidisciplinary teams; data storage and processing; data integration and interpretation; and individual and global economic relevance. This review provides an update of important developments in the analysis of big data and forward strategies to accelerate the global transition to personalized medicine.

## Introduction

Access to large omics (genomics, transcriptomics, proteomics, epigenomic, metagenomics, metabolomics, nutriomics, etc.) data has revolutionized biology and has led to the emergence of systems biology for a better understanding of biological mechanisms. Systems biology aims to model complex biological interactions by integrating information from interdisciplinary fields in a holistic manner (holism instead of the more traditional reductionism). In contrast to treating a mixture of factors as single entities leading to an endpoint, systems biology relies on experimental and computational approaches in order to provide mechanistic insights to an endpoint [1]. Traditional observational epidemiology or biology alone are not sufficient to fully elucidate multifaceted heterogeneous disorders and this directly limits all prevention and treatment pursuits for such diseases [2, 3]. It is widely recognized that multiple dimensions must be considered simultaneously to gain understanding of biological systems [4]. Systems approaches are driving the leading-edge of biology and medicine [5, 6]. The use of deterministic networks for normal and abnormal phenotypes are thought to allow for the proactive maintenance of wellness specific to the individual, that is predictive, preventive, personalized, and participatory medicine (P4, or more generally speaking, personalized medicine) [1].

Many predict the emergence of personalized medicine in the near future, but it is not likely to come about as quickly as the scientific community and the media may think [7]. In parallel to an escalating two-tiered health system at the global level, a similar two-tiered phenomenon is observed with regard to our ability to generate and analyze omics data that may delay even further the transition to personalized medicine. The generation and management (storage, and computational resources) of omics data remain expensive despite technological progress. This implies that personalized medicine could be restricted to the wealthier countries [8]. This is mirrored by a growing gap in our abilities to generate and interpret omics data. The bottleneck in omics approaches is becoming less and less about data generation and more and more about data management, integration, analysis, and interpretation [9]. There is an urgent need to bridge the gap between advances in high-throughput technologies and our ability to manage, integrate, analyze, and interpret omics data [10–12]. This review addresses the growing gaps in socioeconomic and scientific progress toward personalized medicine.

## Review

### The rich get richer and the poor get poorer

**Fig. 1:**
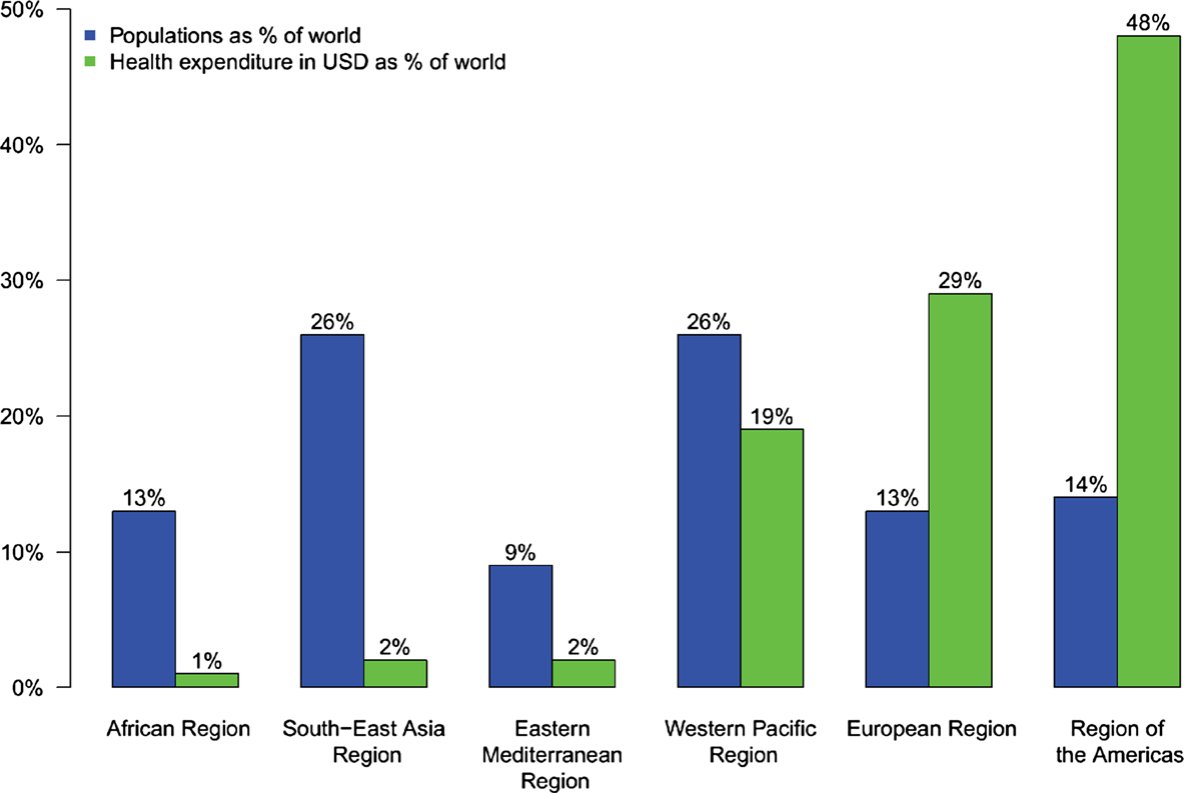
Distributions of populations and global health expenditure according to WHO 2012

The developing world is home to 84 % of the world’s population, yet accounts for only 12 % of the global spending on health [13]. There is a large disparity between the distribution of people and global health expenditures across geographical regions (Fig. 1). While public financing of health from domestic sources has increased globally by 100 % from 1995 to 2006, a majority of low and middle-income countries experienced a reduction of funding during the same time [14]. Several life-threating but easily preventable or treatable diseases are still prevalent in developing countries (e.g. malaria). Personalized medicine will further increase these disparities and many low and middle-income countries may miss the train of personalized medicine [15–17], unless the international community devotes important efforts towards strengthening health systems of the most disadvantaged nations.

Systems medicine, the application of systems biology to human diseases [18], requires investments in infrastructures with cutting-edge omics facilities and analytical tools, advanced digital technologies (high computing performance and storage resources), and highly-qualified multi-disciplinary teams (clinicians, epidemiologists, biologists, computer scientists, statisticians and mathematicians) in addition to investments in security and privacy. On the bright side, technology is evolving quickly and new developments are producing data more efficiently. A few examples include the development of high-throughput next generation sequencing and microarrays in genomics and transcriptomics, mass spectrometry-based flow cytometer in proteomics, real-time medical imaging, and more recently, lab-on-a-chip technologies [19]. Some predict that a technological plateau may be reached for different reasons (reliability, cost-effectiveness), but these projections are not validated by historical trends in science as novel technological developments can always occur [20]. However, there is a consensus that most of the cost in omics studies will come from data analysis rather than data generation [9].

The economic value of omics networks as personalized tests for future disease onset or response to specific treatments / interventions remains largely unknown. A recent study by Philips *et al.* reflects this issue and highlights a lag between clinical and economical value assessment of personalized medical tests in current research [21]. Very few studies have incorporated an economic aspect in the evaluation of personalized tests. These tests range from those available in clinical use or in advanced stage of development, genetic tests with Food and Drug Administration labels, tests with demonstrated clinical utility, and tests examining conditions with high mortality or high health-associated expenditures. Economic evaluations of personalized tests are needed to guide investments and policy decisions. They are an important pre-requisite to hasten the transition to personalized medicine. In addition, those few personalized tests that included economic information were found to be relatively cost-effective, but only a minority of them were cost-saving, suggesting that better health is not necessarily associated with lower expenditures [21]. In summary, the costs associated with personalized medicine transition remain unclear, but personalized medicine may further widen the economic inequality in health systems between high and low-income countries. This jeopardizes social and political pillars of stability, and highlights the need for a broader translation-oriented focus across the globe [22].

Several ideas for stimulating sustainable innovations in developing nations include micro-grants as proposed by Ozdemir V. *et al.* [23]. Although $1,000 micro-grants are relatively small, they far exceed the annual income of individuals below the poverty line of $1.25/day as defined by the World Bank. Recipients of these grants may go a long way in connecting and co-producing knowledge based innovations to broaden translational efforts. Type 1 micro-grants which are awarded through funding agencies may support small labs and local scholars to connect personalized medicine with new models of discovery and translation [23]. Type 2 micro-grants funded by science observatories and/or citizens through crowd-funding mechanisms may facilitate developments of global health diplomacy to share novel innovations (i.e. therapeutics, diagnostics) in areas with similar burdens [23]. There is an overall need to support local scholars in promoting knowledge and innovation within low and middle-income countries [24]. This includes for example, the case of advocating for treatment of persons with Human Immunodeficiency Virus (HIV) infections where their peers may not recognize their illness as an endemic that affects society [24]. One successful example of personalized medicine for HIV patients in low and middle-income countries include personal text messages for improving adherence to antiretroviral therapy in Kenya and Cameroon [25].

Interdisciplinary programs for global translational science such as the Science Peace Corps are another promising catalyzing agent for research and developments in low and middle-income countries (http://www.peacecorps.gov/) [22]. The present Peace Corps program entails volunteer work (6 weeks minimum and up to 2 years) in various regions across the globe to serve as a steady flux of knowledge for translational research. Junior or senior scientists may cover topics from life sciences, medicine, surgery, and psychiatry. This program is bi-directional as it serves both the rich and poor to elucidate the concept of “health” and integrate personalized medicine within various environments. Lagging developments in low and middle-income countries are in fact open opportunities with rewards for intellectual individuals given the simple fact that it is where the majority of the human populations reside.

The “tragedy of the commons” is a conceptual economic problem where the benefits of common and open resources are jeopardized by individuals’ self-interest to optimize personal gains [26]. The 2009 Economics Nobel Laureate, Elinor Ostrom, has shown that this issue is not actually common among humans since individuals work through establishing trust, and tend to find solutions to common problems themselves [27]. Societies do systematically develop complex sustainable regulations to collectively benefit each other where assurance is a critical factor for cooperation [28]. There is a need to understand institutional diversity if humans are to act collectively to benefit each other. Diverse applications of personalized medicine can be envisioned to cope with the diversity of the world by allowing multi-tier personalized health care systems at multiple scales and avoiding a single top-tier health care system that may instead compromise resource management. This also brings about the need for nested regulation systems for both science and ethics (i.e. ethics-of-ethics) as the assurance factor for cooperation [29, 30]. Transparency and accountability need to be imposed on all scientists, practitioners, ethicists, sociologists, and policymakers. No one should be above the fray for accountability if a sustainable transition towards personalized medicine is to occur.

### Omics data: the shifting bottlenecks

In parallel to the gap in health systems between rich and poor countries that personalized medicine may widen, an increasing lag has been observed in our ability to generate *versus* integrate and interpret omics data these last ten years [9]. New technologies and knowledge emerging from the Human Genome Project, fueled by biotechnology companies, led to the omics revolution in the beginning of the 21^th^ century [31]. Using high-throughput technologies, we are now able to perform an exhaustive number of measurements over a short period of time giving access to individuals’ DNA (genomics), transcribed RNA from genes over time (transcriptomics), DNA methylation and protein profiles of specific tissues and cells (epigenomics and proteomics), metabolites (metabolomics), among other types of omics data [32]. Even histopathological and radiological images which are traditionally evaluated and scored by trained experts are now subjected to computational quantifications (i.e. imaging informatics) [10–12, 33]. Business models based on returns on investments have driven ongoing technological developments to accelerate the generation of omics data at increased affordability in comparison with existing technologies. As a consequence, omics platforms and individual omics profiles are expected to become fairly affordable and data generation is no more a bottleneck for most laboratories, at least in the middle and high-income countries [34].

**Fig. 2:**
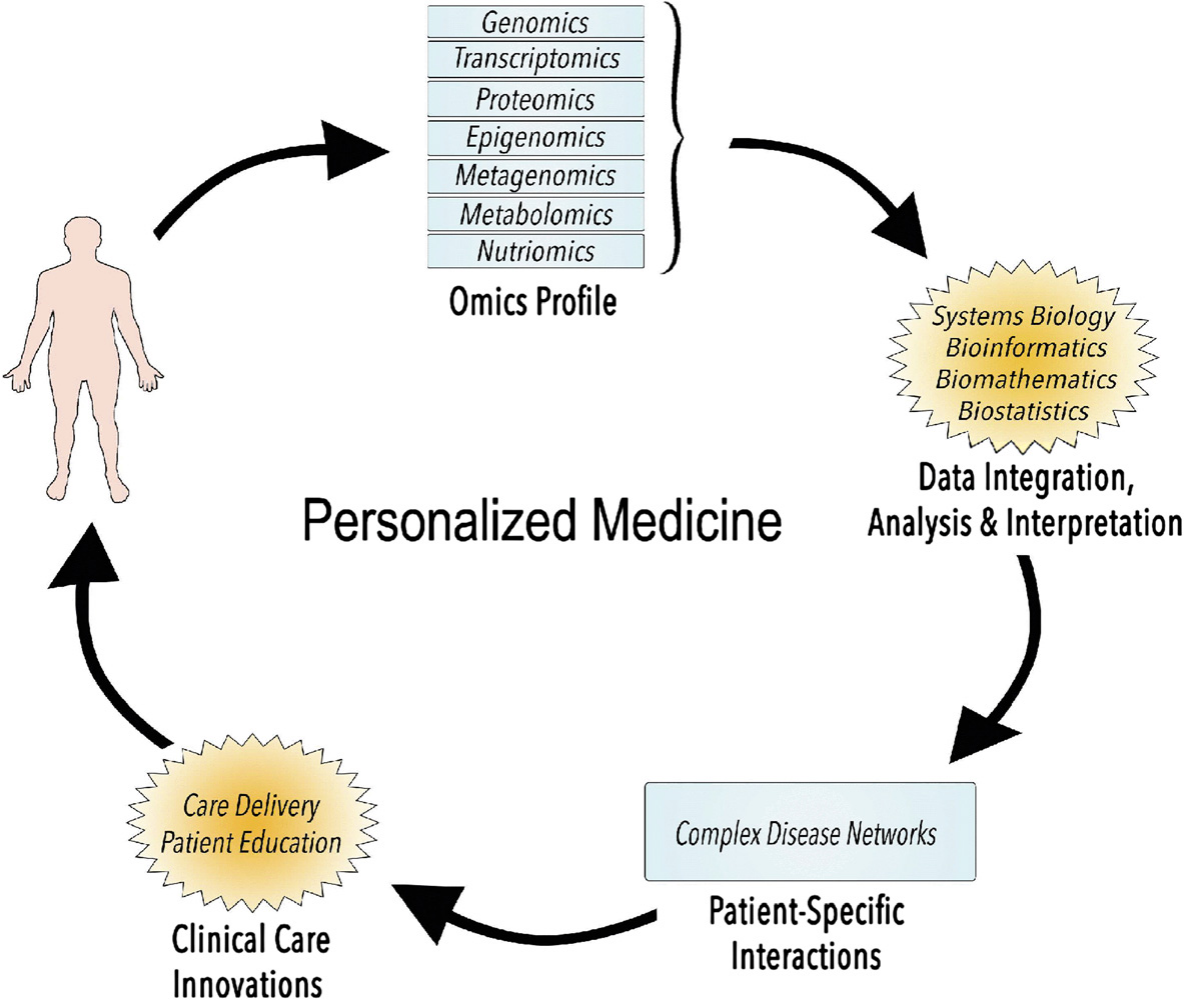
A basic framework of personalized medicine. The integration of omics profiles permit accurate modeling of complex diseases and opens windows of opportunities for innovative clinical applications to subsequently benefit the patient

Initially, there were great expectations for omics data to provide clues on the mechanisms underlying disease initiation and progression as well as new strategies for disease prediction, prevention and treatment [1]. The idea was to translate omics profiles into subject-specific care based on their disease networks (Fig. 2). However, our ability to decipher molecular mechanisms that regulate complex relationships remains limited despite growing access to omics profiles. Biological processes are very complex, and this coupled with the noisy nature of experimental data (e.g. cellular heterogeneity) and the limitations of statistical analyses (e.g. false positive associations) poses many challenges to detecting interactions between “networks” and “networks of networks”. As an illustration, only a minority of the genetic variants predisposing to type 2 diabetes have been identified so far, despite large-scale studies involving up to 150,000 subjects [1, 35]. It becomes more and more obvious that the bottleneck in laboratories has shifted from data generation to data management and interpretation [36].

### Personalized medicine needs hybrid education

Although solutions for the challenges of big data already exist and are adopted by companies such as Google, Apple, Amazon, and Facebook to tackle the fairly homogenous big data (i.e. user data) [37], the heterogeneous nature of omics data presents a new challenge that requires sufficient understanding of the underlying biological concepts and analysis algorithms to carry out data integration and interpretation [38]. It is important for the working scientist to understand 1) the underlying problem, 2) the methods of data analysis, and 3) the advantages, and disadvantages of different computational platforms to carry out explorations and draw inference. Expertise in biology provides a foundation to contextualize causal effects and guide identification and interpretation of interaction signals from noise. There is also no uniformly most powerful method to analyze omics data and the use of various approaches to infer biological interactions requires modeling expertise [39]. Otherwise, research quality is sacrificed to avoid the logistical challenges of modeling in exchange for the use of more straightforward approaches [40]. Lastly, computer programing skills are necessary to navigate explorations and analyze omics data accordingly. There is a need for reliable and maintainable computer codes through best practices for scientific computing [41]. Approximately 90 % of scientists are self-taught in developing software and one may lack basic practices such as task automation, code review, unit testing, version control and issue tracking [42, 43]. Barriers between disciplines still exist between informaticians, mathematicians, statisticians, biologists, and clinicians due to a too divergent scientific background. Cutting-edge science is integrative by essence and innovative strategies in universities to educate and train future researchers at the interface of traditionally partitioned disciplines is urgently needed for the transition to personalized medicine. Johns Hopkins University is leading this evolution by changing the teaching plans and establishing new programs in the school of medicine that integrate the notion of personalized medicine [44]. Although increased knowledge at the population level is a key factor in development of modern societies, there is an upper limit to the wealth of knowledge and expertise a single individual can hold [45]. This is the reason why, in addition to multidisciplinary individual training, initiatives by universities, research funding agencies, and governments are encouraged to connect researchers from diverse scientific backgrounds on interface topics related to systems biology and personalized medicine. The recent shift by the Canadian Institutes of Health Research from distinct discipline (e.g. genetics) to multidisciplinary expert panels in funding biomedical research is a step in the right direction. The creation of interdisciplinary research institutes, such as the Steno Diabetes Center in Denmark that combine clinical, educational and multifaceted research activities to lead translational research in diabetes care and prevention, is another sensible initiative that could prefigure what may become personalized medicine institutes in the future.

### Management and processing of omics data

**Fig. 3:**
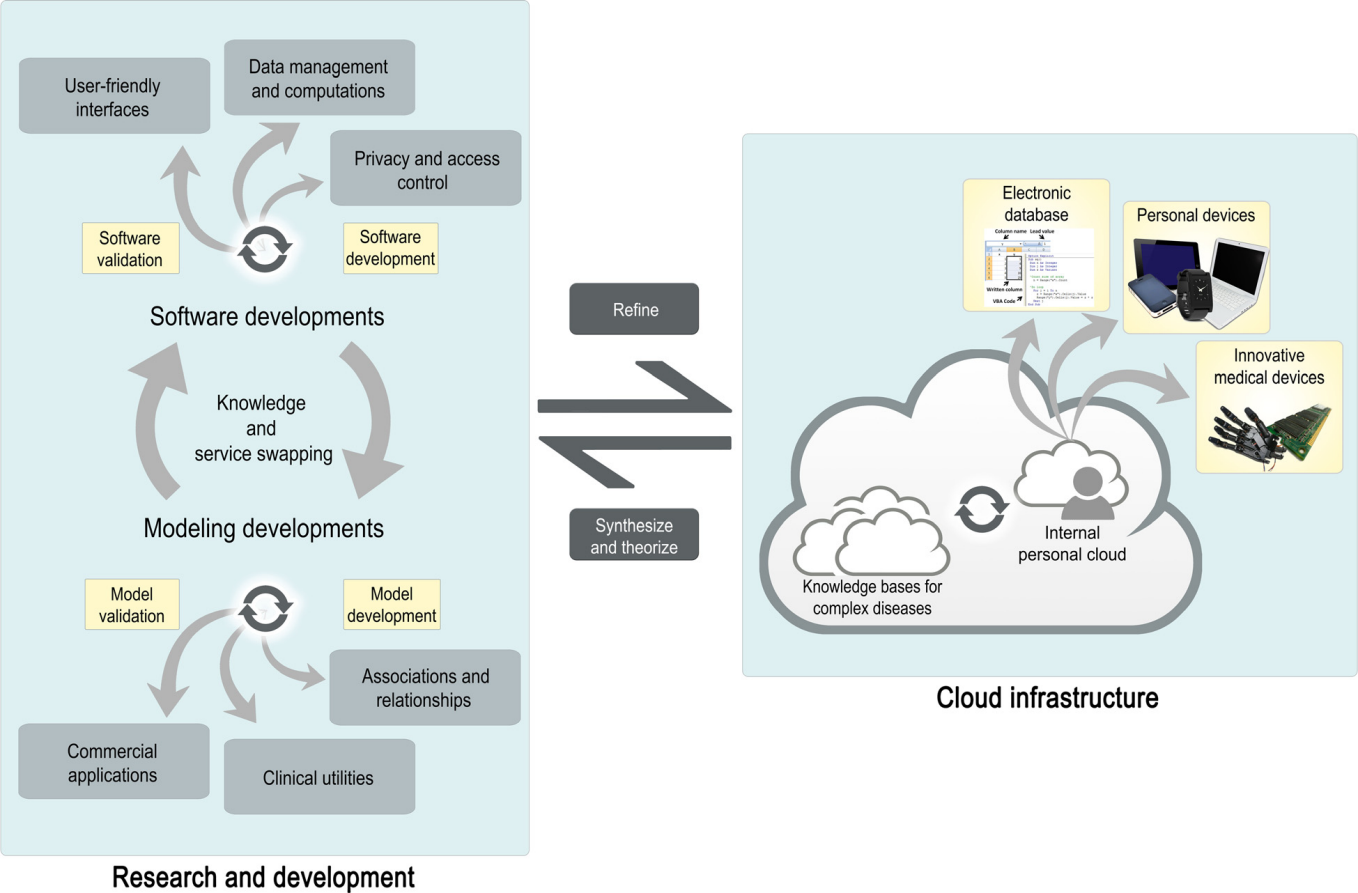
An interdisciplinary cloud-based model to implement personalized medicine. The consecutive knowledge and service swapping between modeling and software experts in research and development units is essential for the management, integration, and analysis of omics data. Thorough software and model development will derive updates upon knowledge bases for complex diseases, in addition to clinical utilities, commercial applications, privacy and access control, user-friendly interfaces, and advanced software for fast computations within the cloud. This translates into personalized medicine *via* personal clouds that upload wellness indices into personal devices, electronic databases for health professionals, and innovative medical devices

Major investments need to be made in bioinformatics, biomathematics, and biostatistics by the scientific community to accelerate the transition to personalized medicine. Classic research laboratories do not possess sufficient storage and computational resources for processing omics data. Laboratory-hosted servers require investments in informatics support for configuring and using software. Such servers are not only expensive to setup and maintain, but do not meet the dynamic requirements of different workflows for processing omics data, leading to either extravagant or sub-optimal servers. One promising technology to close the gap between generation and handling of omics data is cloud computing [46, 47]. It is an adaptive storage and computing service that exploits the full potential of multiple computers together as a virtual resource *via* the Internet [48]. Examples include the EasyGenomics cloud in Beijing Genomics Institute (BGI), and “Embassy” clouds as part of ELIXIR project in collaboration with multiple European countries (UK, Sweden, Switzerland, Czech Republic, Estonia, Norway, the Netherlands, and Denmark) [49]. The focus is currently placed on developing cloud-based toolkits and workflow platforms for high-throughput processing and analysis of omics data [50, 51, 49, 52]. More recently, Graphics Processing Units (GPUs) have been proposed for general-purpose computing in a cloud environment [53]. GPUs provide faster computations as accelerators by one or two orders of magnitudes compared to general Central Processing Units (CPUs) and have been exploited to cope with exponentially growing data [54–56]. MUMmerGPU for example, processes queries in parallel on a graphics card, achieves more than a 10-fold speedup over a CPU version of the sequence alignment kernel, and outperforms the CPU version of MUMmer by 3.5-fold in total application time when aligning reads [57]. However, a significant amount of work will be required for developing parallelization algorithms considering the heterogeneous framework of omics data that present challenges in communications and synchronizations [37]. There are tradeoffs between computational cost (floating-point operations), synchronization, and communications to consider while developing parallelization algorithms [58]. Moreover, developing error-free and secure applications is a challenging and labor-intensive, yet critically important task. Examples of programming errors and studies outlining wrongly mapped SNPs in commercial SNP chips have been reported in literature [59–61]. There is a need to validate the reliability of research platforms before considering the clinical utility of omics data. For instance, ToolShed, a feature of the Galaxy project that draws in software developers worldwide to upload and validate software tools, aims to enhance the reliability of bioinformatics tools. Novel tools and workflows with demonstrated usefulness and instructions are publically available (http://toolshed.g2.bx.psu.edu/) [62]. Both storage and computing platform such as Bioimbus [63], Bioconductor [64], CytoScape [65], are made available by scientists to exchange algorithms and data. There are many questions and methodologies that researchers may wish to consider, and this continuously drives on novel bioinformatics tools. Ultimately, lightweight programing environments and supporting programs with diverse cloud-based utilities are essential to enable those without or with limited programing skills to investigate biological networks [66]. Figure 3 illustrates a cloud-based framework that may help to implement personalized medicine. Much more programing efforts are still needed for the integration and interpretation of omics data in the transition to personalized medicine. Potential downstream applications are not always apparent when data are generated, promoting sophisticated flexible programs that may be regularly updated [67].

### Integrative methods of omics data

Lastly, the depiction of biological systems through the integration of omics data requires appropriate mathematical and statistical methodologies to infer and describe causal links between different subcomponents [40]. The integration of omics data is both a challenge and an opportunity in biostatistics and biomathematics that is an increasing reality with the decreasing costs of omics profiles. Aside from the computational complexity of analyzing thousands of measurements, the extraction of correlations as true and meaningful biological interactions is not trivial. Biological systems include non-linear interactions and joint effects of multiple factors that make it difficult to distinguish signals from random errors. Caspase-8 for example, has opposing biological functions as it promotes cell death by triggering the extrinsic pathway of apoptosis, while having beneficial effects on cell survival through embryonic development, T-lymphocyte activation, and resistance to necrosis induced by tumor necrosis factor-α (TNF-α) [68]. Genes may carry out different functions in different cell types / tissues, which adds to the already substantial inter-individual variability. Biological complexity presents a challenge in extracting useful information within high-dimensional data [69]. Both computational and experimental methodologies are needed to fully elucidate biological networks. However, in contrast to experimental assays, computational models rely on biologically driven variables and have inherent pitfalls of omics data.

### Coping with to the curse of dimensionality

**Fig. 4:**
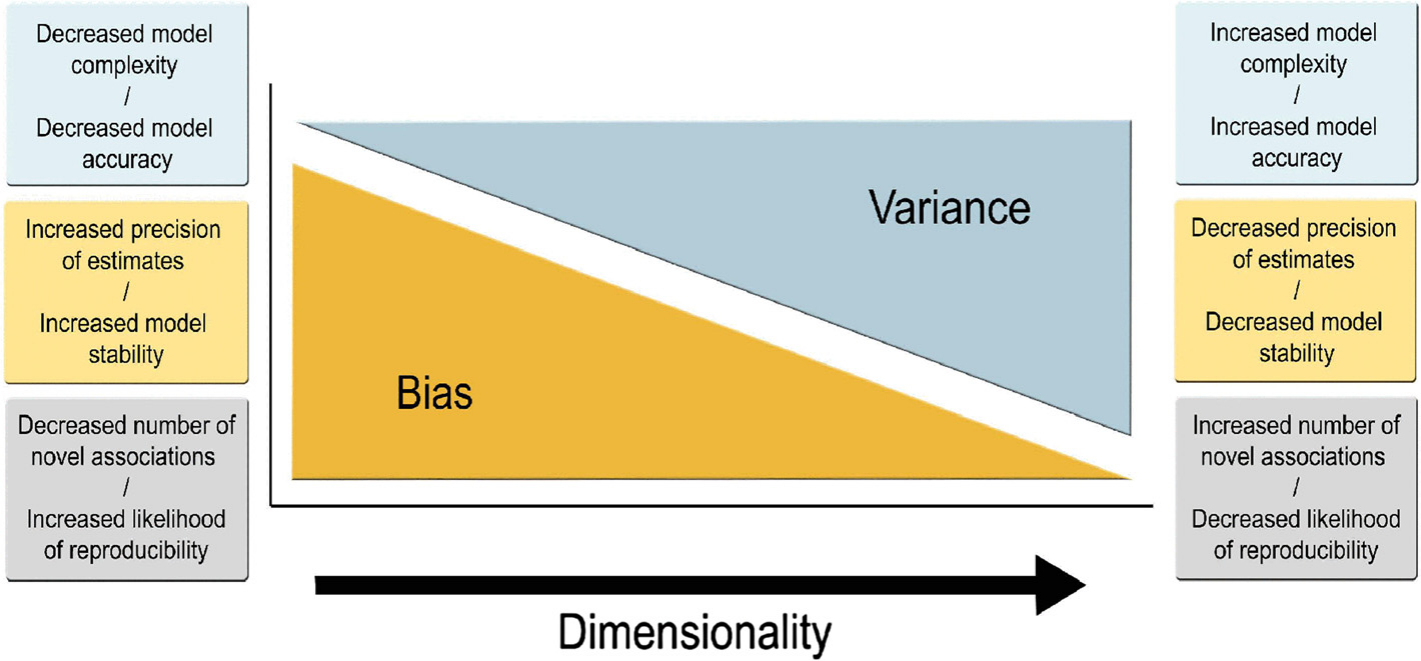
The bias-variance tradeoff with increasing model complexity

High-dimensionality is one of the main challenges that biostatisticians and biomathematicians face when deciphering omics data. It is the issue of “large *p*, small *n*”, where the number of measurements, *p*, is far greater than the number of independent samples, *n* [69, 33]. The analysis of thousands of measurements often leads to results with poor biological interpretability and plausibility. The reliability of models decreases with each added dimension (i.e. increased model complexity) for a fixed sample size (i.e. bias-variance dilemma, see Fig. 4) [69]. All estimate instability, model overfitting, local convergence, and large standard errors compromise the prediction advantage provided by multiple measures. A better understanding of these inherent caveats comes from the key concept behind statistical inference that is the distribution of repeated identical experiments. This distribution can be characterized by parameters such as the mean, and variance that quantify the average value (i.e. effect size), and degree of variability (i.e. biological or experimental noise). These parameters are estimated from observed data drawn from the true distribution (i.e. a finite number of independent samples). The reliability of estimates from a small sample size is low where it is more likely to observe estimates that deviate from the true distribution parameters. The chance of encountering such deviations also increases with the number of different measurements in a fixed sample. It is difficult to reliably estimate many parameters, and correctly infer associations from multiple hypotheses tested simultaneously. As a result, the analysis of both single and integrative omics data is prone to high rates of false-positives due to chance alone. This requires researchers to adjust for multiple testing to control for type 1 error rate using various methods based on the family-wise error rate (e.g. Bonferroni corrections, Westfall and Young permutation), and the false-positive rate (e.g. Benjamin and Hochberg) that are under strict assumptions [70–75]. Another solution to overcome multiple testing issues is to reduce dimensionality *via* sparse methods that provide sparse linear combinations from a subset of relevant variables (i.e. sparse canonical correlation analysis, sparse principal components analysis, sparse regression) [76, 77]. Both *mixOmics* and *integrOmics* are publically available R packages for utilizing sparse methods on omics data [77, 78]. There are several approaches to derive “optimal” tuning parameters to dictate the number of relevant variables to pursue [79, 80]. However, stochastic processes to select “best” subsets of variables inferred from a given sample population may not contain the best information on another independent study, and certainly not at an individual level (i.e. selection-bias) [81, 82]. Reducing dimensionality is problematic as key mechanistic information could be lost. There is an overall tradeoff between false positive rates and the benefit of identifying novel associations within biological process that align with that of bias and variance (Fig. 4) [70].

The multi-level ontology analyses (MONA) is one approach that bypasses the high-dimensionality as described by Sass *et al.* [83]. This method integrates multiple omics information (DNA sequence, mRNA and protein expressions, DNA methylation, and other regulation factors) and copes with redundancies related to multiple testing problems by approximating marginal probabilities using the expectation propagation algorithm [84]. The MONA approach allows for biological insights to be incorporated into the defined network as prior knowledge. This can address overfitting or uncertainty issues though reducing the solutions space to biological meaningful regions [85, 86]. This approach, however, relies on predefined known biological networks (i.e. protein–protein interactions) or on the accuracy of mechanistic models (i.e. network models). Another strategy to analyze omics data involves integrating multiple data types into one single data set that holds maximum information. This reduces the complexity of omics data to the analysis of a single high-dimensional data set. Co-inertia analysis for example, has been used to integrate both proteomic and gene expression data to visualize and identify clusters of networks [87, 88]. It was initially introduced by Culhane *et al.* to compare gene expression data provided by different platforms, but has been further generalized to assess similarities between omic data sets [89]. The basic principal is to apply within and between principal component analysis, correspondence analysis, or multiple correspondence analysis while maximizing the sum of squares of covariances between variables (i.e. maximizing co-inertia between hyperspaces). The *omicade4* package in R is available for exploring omics data using multiple co-inertia analysis [90]. Other similar, but conceptually different approaches include generalized singular value decomposition [91], and integrative bioclustering methods [92, 93]. An integrative omics study by Tomescu *et al.*, have utilized all three approaches to characterize networks within *Plasmodium faclicparum* at different stages of life cycles [94]. Although the basic mathematical assumptions are different, the overlap in their results was considerable. The relative importance and incremental value of individual omics data on one another may also be considered when predicting specific outcomes. For instance, Hamid *et al.* recently proposed a weighted kernel Fisher discriminant analysis that accounts for both quality and informativity of each individual omics data to integrate [95]. Significant improvements however, may not occur when data are redundant (i.e. correlated) or of low quality.

### Mixing apples and oranges

**Fig. 5:**
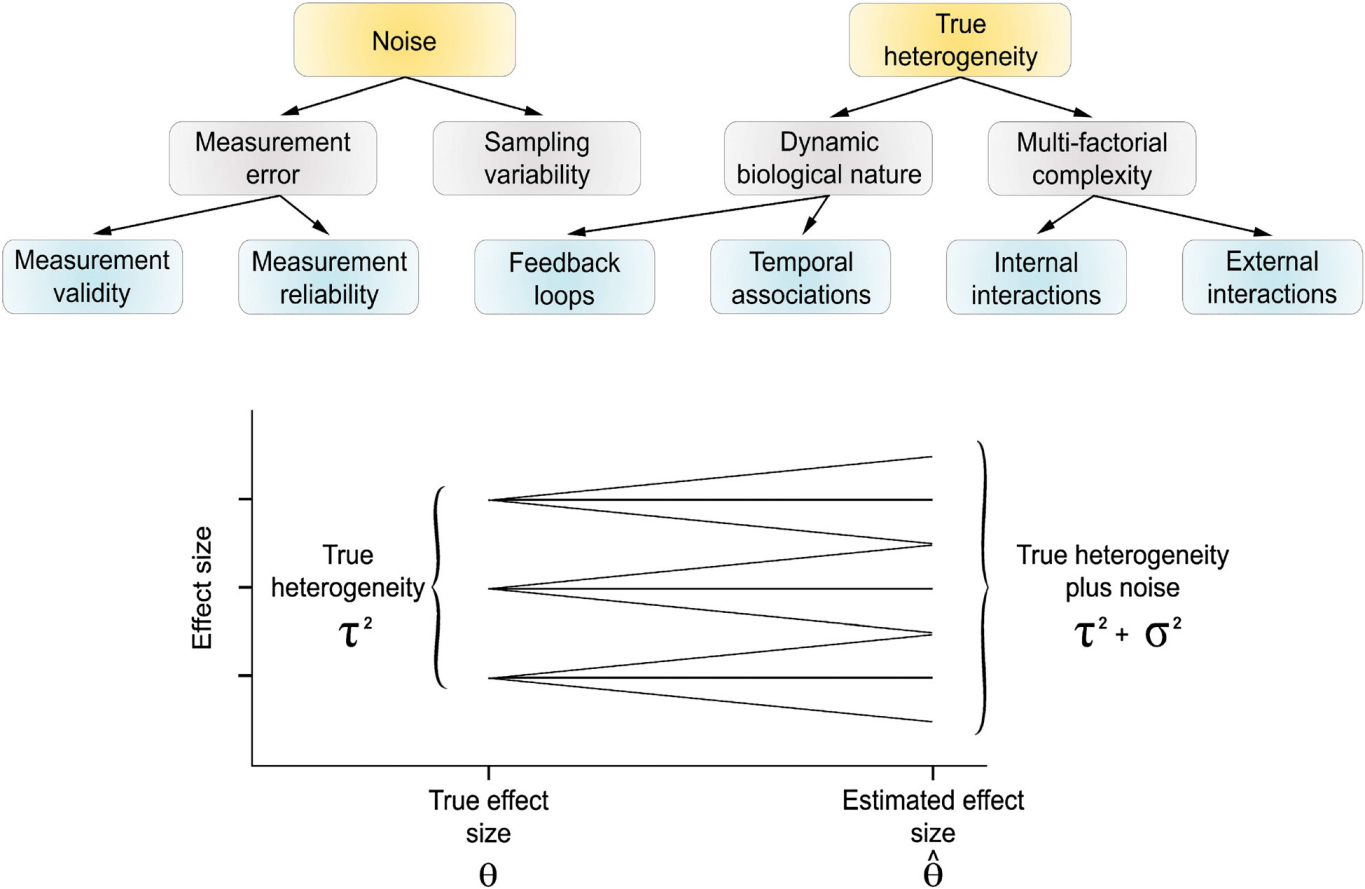
Noise and true heterogeneity within complex systems. Source of noise include measurement error and sampling variability. True heterogeneity however, is the result of true differences of effect sizes due to 1) the dynamic biological nature which encompasses feedback loops and temporal associations; and 2) multi-factorial complexity. Increasing the sample sizes is one solution to bypass noise and attain precise effect sizes, but true heterogeneity can only be adjusted during analysis when possible and *via* standardizations and calibrations that limit generalizability of the conclusions

Another challenge for integrating omics data lies in deriving meaningful interpretable correlations. For example, direct correlation analyses between transcriptomics and proteomics profiles are not valid in eukaryotic organisms. No high correlations between the two domains were observed as reported by multiple studies, and this was attributed to post-transcriptional and post-translational regulations [96–99]. The advantage of integrating transcriptomic and proteomic data may diminish without accounting for regulation factors as the resulting inflated variability may limit reliability and reproducibility of findings [100]. Many complex traits are tightly regulated and incorporating regulation factors may explain a relevant portion of observed variations due to true heterogeneity (i.e. true differences in effect sizes). Unlike the impact of noise on estimate precision which could be minimized by increasing the sample size, true heterogeneity may only be adjusted for during analysis when possible or *via* standardizations that limit generalizability. True heterogeneity poses a problem given biological complexity in the pursuit of precise effect size estimations (Fig. 5). Hence, there is a need for network analysis to account for protein-protein and protein-DNA interactions in the context of integrating transcriptomics and proteomics data alone. An early study by Hwang *et al.* utilized network models to identify protein-protein and DNA-protein interactions with experimental verifications [101].

Bayesian networks are graphical models that involve structure and parameter optimization steps to represent probabilistic dependencies [102]. This modeling strategy that elucidates biological networks has been utilized in various studies [103, 104]. A seminal example includes the use of dynamic Bayesian networks trained on chromatin data to identify expressed and non-expressed DNA segments in a myeloid leukemia cell line [105]. This was done by integrating position of histone modifications, and transcription factors’ binding sites at multiple intervals. It is however, a computationally demanding approach that requires advanced computing methods such as parallel computing and acceleration *via* GPUs [106]. Network models may serve as meaningful statistical results to be integrated with the biological domain. It has the potential to generate insight and a number of hypotheses on biological interactions to be experimentally and/or independently verified through a follow-up validation set. The ultimate goal is to continuously provide insight into biological interactions to subsequently build upon.

### Separate the wheat from the chaff

It is important to minimize sources of error with omics data as it is challenging to distinguish between random error and true interaction signals. Hence, it is necessary to utilize statistical methods to account for sources of error. For example, the quality of omics data may vary between high-throughput platforms. Hu *et al.* have proposed quality-adjusted effect size models that were used to integrate multiple gene-expression microarray data given heterogeneous microarray experimental standards [107]. Omic studies are also prone to errors such as sample swapping and improper data entry. New methodologies for assessing data quality include Multi-Omics Data Matcher (MODMatcher) [108]. Moreover, complex diseases are often evaluated using a single phenotype that compromises statistical analysis by introducing errors such as misclassifications, and/or lack of accountability for disease severity [109]. Modeling images for example, requires multiple phenotypes to properly capture image features [110]. Joint modeling of multiple responses to accurately capture complex phenotypes has been shown to increase power of discovery in genome-wide association studies [111]. There are even novel network methodologies to account for within-disease heterogeneity [112, 113]. Network approaches in modeling complex diseases may provide a map of disease progression and play a major role in the proactive maintenance of wellness [114]. All reproducibility and validations of complex interaction signals are essential in the pursuit of personalized medicine. This highlights the growing need for metadata as the science of hows (i.e. “data about data”) to help harmonize omics studies and enable proper reproducibility of research results [115]. Examples of a metadata checklist and a metadata publication are available [116, 117]. Metadata may also serve as open innovations for integrative sciences, and may prove to be valuable for diversifying models of discovery and translation in high, and more importantly, low and middle-income countries. Altogether, validations on multiple data sets are required as evidence of stability, and that theoretically sound new methods outperform existing ones [118]. Both descriptive and mechanistic models for determining relevant biological networks require handling with care [119]. Software that integrate and interpret omics data are currently developed by competing companies in the private sector (e.g. Anaxomics, LifeMap), which may rapidly advance the field in the near future.

## Conclusion

**Fig. 6:**
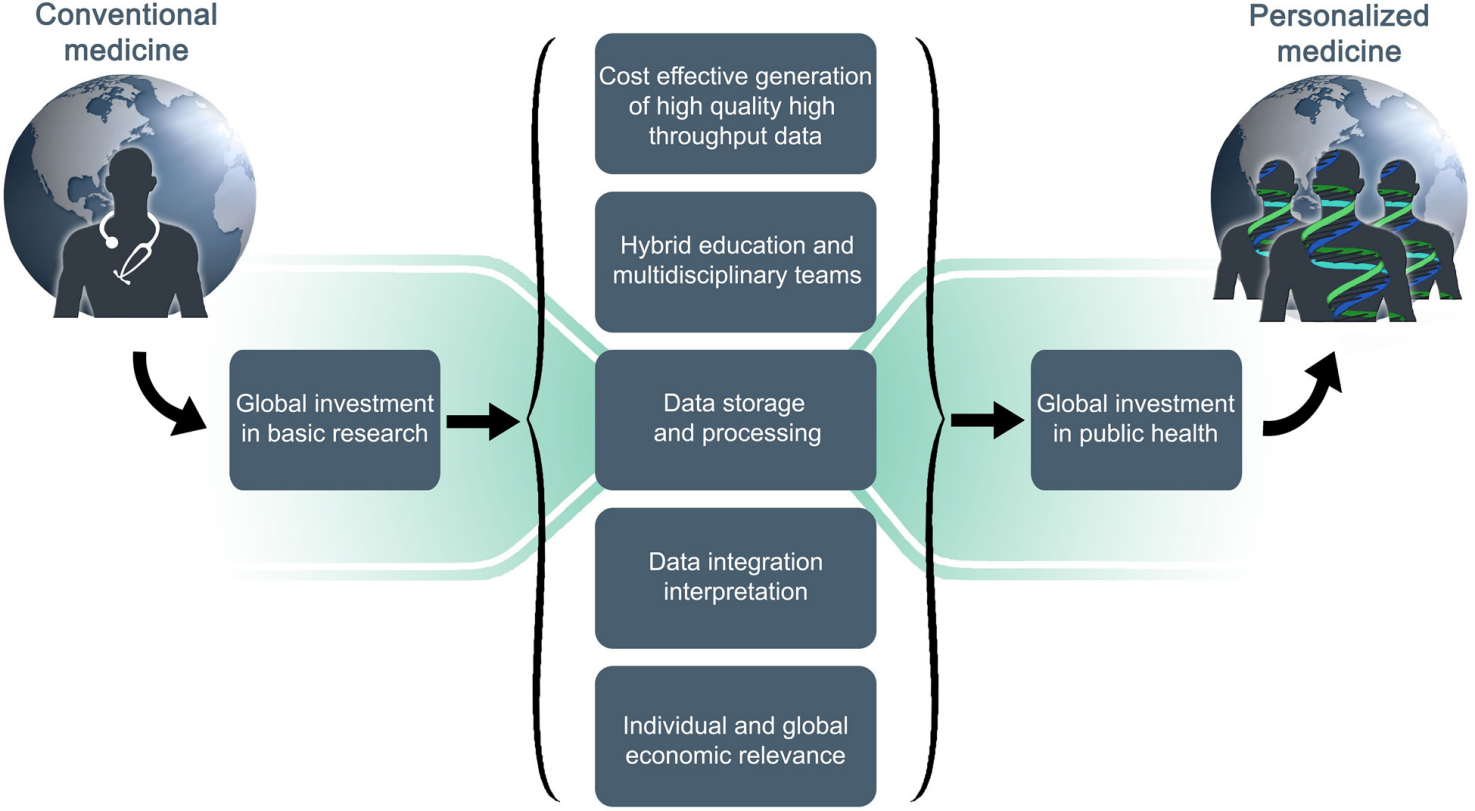
Bottleneck toward personalized medicine. The collective challenges to make the transition from conventional to personalized medicine include: i) generation of cost-effective high-throughput data; ii) hybrid education and multidisciplinary teams; iii) data storage and processing; iv) data integration and interpretation; and v) individual and global economic relevance. Massive global investment in basic research may precede global investment in public health for transformative medicine

This review aims to stimulate research initiatives in the field of big data analysis and integration. Omics data embody a large mixture of signals and errors, where our current ability to identify novel associations comes at the cost of tolerating larger error thresholds in the context of big data. Major investments need to be made in the fields of bioinformatics, biomathematics, and biostatistics to develop translational analyses of omics data and make the best use of high-throughput technologies. New generations of multi-talented scientists and multidisciplinary research teams are required to build accurate complex disease models and permit effective personalized prevention, diagnosis and treatment strategies. Our ability to integrate and interoperate omics data is an important limiting factor in the transition to personalized medicine. Overcoming these limitations may boost the nation-wide implementation of omics facilities in clinical settings (Fig. 6). The subsequent economies of scale may in turn favor the access to personalized medicine to disadvantaged nations, repelling the growing shadow of two-tiered personalized medicine.
